# Short prolactin isoforms are expressed in photoreceptors of canine retinas undergoing retinal degeneration

**DOI:** 10.1038/s41598-020-80691-6

**Published:** 2021-01-11

**Authors:** Raghavi Sudharsan, Leonardo Murgiano, Hsin-Yao Tang, Timothy W. Olsen, Venkata R. M. Chavali, Gustavo D. Aguirre, William A. Beltran

**Affiliations:** 1 Division of Experimental Retinal Therapies, Section of Ophthalmology, Department of Clinical Sciences and Advanced Medicine, School of Veterinary Medicine, University of Pennsylvania, 3900 Delancey Street, Philadelphia, PA 19104 USA; 2 Proteomics and Metabolomics Facility, Wistar Institute, Philadelphia, PA 19104 USA; 3 Department of Ophthalmology, Mayo Clinic, Rochester, MN 55905 USA; 4 Department of Ophthalmology, Perelman School of Medicine, University of Pennsylvania, Philadelphia, PA 19104 USA

**Keywords:** Cell biology, Neuroscience, Diseases

## Abstract

Prolactin (PRL) hormone functions as a pleiotropic cytokine with a protective role in the retina. We recently identified by transcriptome profiling that *PRL* is one of the most highly upregulated mRNAs in the retinas of mutant rcd1 (*PDE6B*) and xlpra2 (*RPGR*) dogs at advanced stages of photoreceptor disease. In the present study, we have identified the expression of a short *PRL* isoform that lacks exon 1 in canine retinas and analyzed the time-course of expression and localization of this isoform in the retinas of these two models. Using laser capture microdissection to isolate RNA from each of the retinal cellular layers, we found by qPCR that this short *PRL* isoform is expressed in photoreceptors of degenerating retinas. We confirmed by in situ hybridization that its expression is localized to the outer nuclear layer and begins shortly after the onset of disease at the time of peak photoreceptor cell death in both models. PRL protein was also detected only in mutant dog retinas. Our results call for further investigations into the role of this novel PRL isoform in retinal degeneration.

## Introduction

Hormones control several important aspects of retinal development, differentiation, maturation, and normal visual processes. The role of amino-acid derived hormones (melatonin, dopamine, thyroxine)^1–3^, steroidal hormones (estrogen, progesterone, aldosterone, corticosteroids)^4–6^ and protein/peptide hormones (growth hormone, somatostatin, prolactin, aldosterone)^7–9^ in normal retinal homeostasis and retinal degeneration has been investigated extensively over the past decades. Working through diverse pathways, most hormones modulate retinal cell survival and health by regulating angiogenesis^5,6,9–11^, inflammation^3,5,6,12^, expression of pro-survival, pro-apoptotic and neurotropic factors^4,5,13^, and via their antioxidant actions^3,4,8,14,15^. Receptors and protein binding partners for these hormones are expressed in retina. In addition, a number of these hormones are synthesized (steroid hormones, melatonin) or transcribed and translated (growth hormone, prolactin) in situ by various retinal cells^2,4,5,9,12^.

Prolactin (PRL), a somatolactogenic polypeptide secreted by the lactotrophic cells of the anterior pituitary gland, is one such hormone with homeostatic and protective roles in retina. PRL was initially characterized as a hormone required for lactation and mammary gland development^16^, but is now recognized as a pleiotropic cytokine with a multitude of tissue-specific functions^17,18^. PRL produced by the pituitary gland enters the retina through the circulation^19^, yet its function in the tissue is still a matter of debate. Early studies performed in hypophysectomized rats showed significant reduction in retinal damage triggered by light exposure. This protection could be reversed by introducing PRL back in the circulation, either by systemic injection or implantation of pituitary gland over the kidney capsule^20^. In contrast, in recent studies, PRL signaling in rats was shown to protect from light-induced damage by modulating the neurotrophin secretion from glial cells in the retina^13^. PRL also has a homeostatic and anti-apoptotic role in retinal pigment epithelium (RPE)^15,21^, and functions primarily by activating the JAK-STAT pathway via its ubiquitously expressed PRL receptor (PRLR)^22^. Additionally, PRL is cleaved by proteases such as cathepsin D, matrix metalloproteases and bone morphogenetic protein-1 to generate a family of bioactive peptides, collectively called vasoinhibins^11,23,24^. While PRL is pro-angiogenic^25^, the vasoinhibins have antiangiogenic effects in ocular tissue including the neuroretina^10,25–27^. They have been shown to be protective against diabetic macular edema caused by blood-retinal barrier breakdown^28^, and are being investigated for their protective effect in diabetic retinopathy in an ongoing clinical trial (NCT03161652)^29^.

PRL is also produced by a number of extra-pituitary tissues^30,31^, including the retina^10,32^, and its mRNA was found in all three retinal cellular layers in normal rat retina using RT-PCR and in situ hybridization (ISH)^10,32^. In a recent study that analyzed the transcriptional changes in retina occurring at an advanced disease stage (≥ 50% photoreceptor loss) of two non-allelic canine forms of inherited retinal degeneration (rcd1/*PDE6B* mutation and xlpra2/*RPGR* mutation), we found that *PRL* was one of the top upregulated (50–150 fold) transcripts in both diseases^33^. In the current study, we have further examined the expression of *PRL* mRNA in dog retinas during retinal degeneration in an attempt to understand its potential role in photoreceptor cell survival/death. We now report that photoreceptors in degenerating canine retinas express a novel *PRL* isoform.

## Results

### *PRL* mRNA upregulated in degenerating canine retinas lacks exon 1

**Figure 1:**
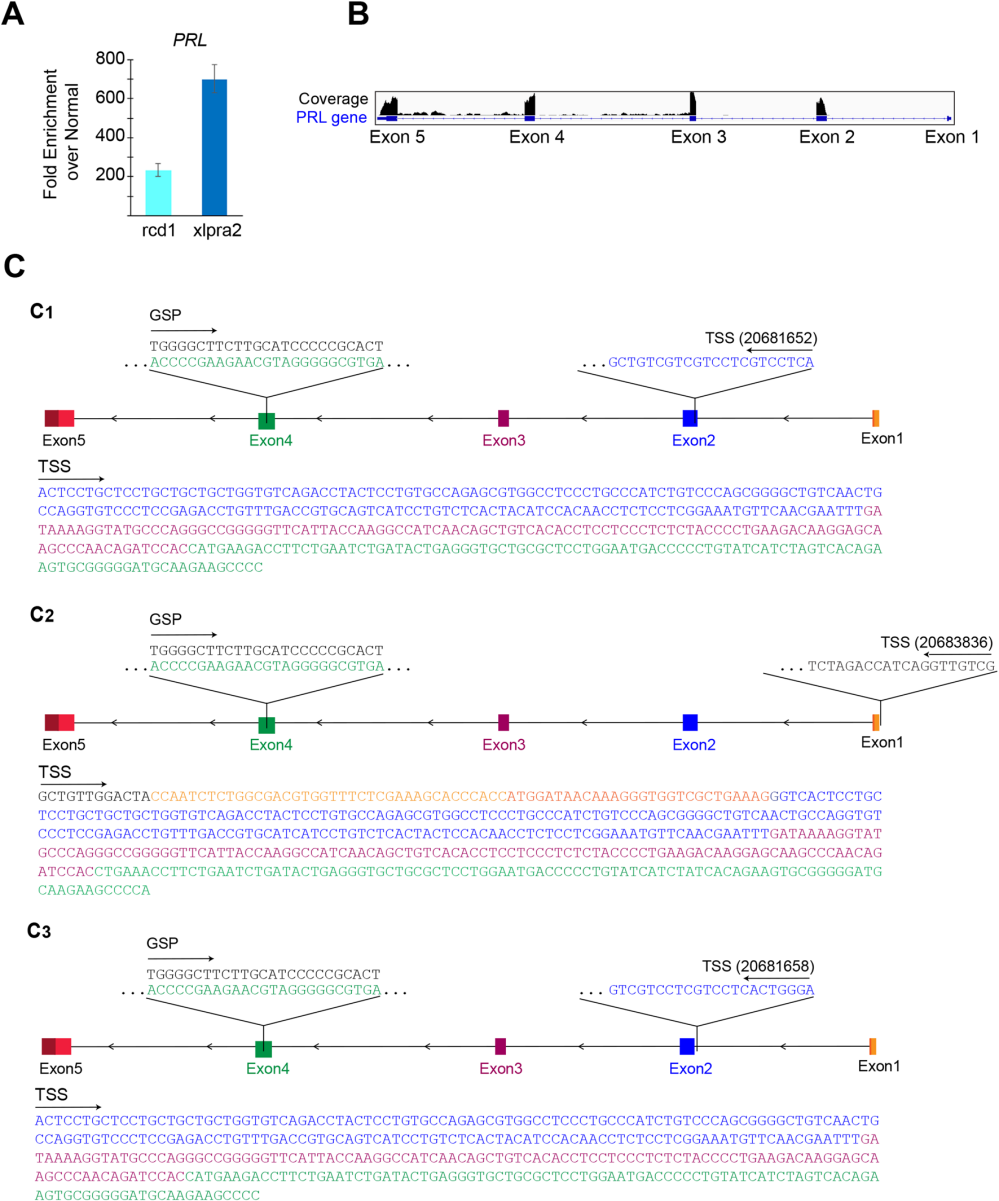
Canine retinal Prolactin isoform lacks exon 1. **(A)** Fold enrichment of *PRL* mRNA in rcd1 and xlpra2 retinas over normal (n = 3 animals per group) (**B)** Representative graph derived from Integrated Genomic View (IGV) showing RNAseq (GSE97638)^33^ reads mapped by STAR for the *PRL* exons expressed in retinas of rcd1 and xlpra2 dogs. The annotated 5 exons present in the reference (CanFam3.1) are shown, however, only exons 2, 3, 4 and 5 show any coverage. No reads were mapped to exon 1. **C)** Transcription start site (TSS) for dog retina (n = 4) **(C1)** and pituitary (n = 2 dogs) **(C2,C3)** *PRL* identified by 5′-RACE. GSP = gene specific primer.

We have validated our previous RNAseq transcriptomic analysis^33^ that compared retinal expression profiles from normal and mutant dogs at advanced stage of retinal degeneration (≥ 50% photoreceptor cell loss) by performing qRT-PCR for *PRL* on RNAs from the same retinal tissues using primers located in exons 2 and 3. We confirmed that *PRL* mRNA was indeed upregulated in both mutant retinas (Fig. 1A). Similar to our published RNAseq results, *PRL* mRNA expression in xlpra2 retinas was higher than in rcd1 retinas.

**Figure 2:**
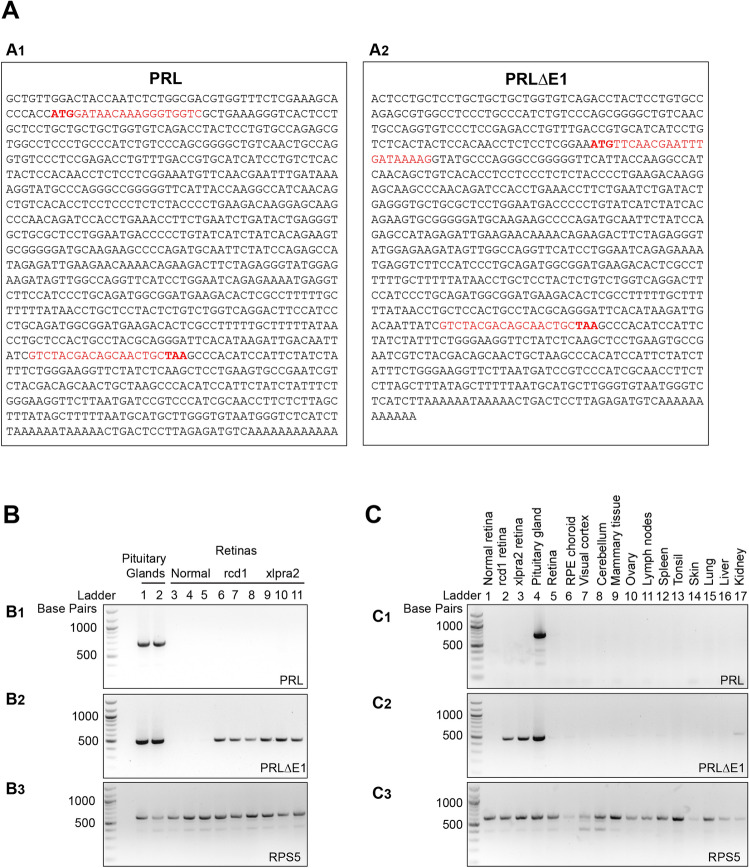
Expression of full length and short *PRL* transcripts in canine tissues.** (A)** Nucleotide sequences of full length **(A1)** and short **(A2)** canine *PRL* isoforms with positions of the translational start (ATG) and end (TAA) sites, and forward and reverse primers (red) used for PCR amplification. (**B)** PCR amplification of *PRL* transcripts from pituitary gland and retinas (normal and mutant). The full length *PRL* transcript is found in pituitary gland (n = 2 dogs) but not in retina (n = 3 dogs per group) **(B1)**, whereas the short *PRL* isoform is found only in mutant retinas **(B2)**. Amplification of housekeeping gene RPS5 was included as a control **(B3)**. (**C)** Analysis of *PRL* mRNA expression in various tissues from a normal dog. Full length *PRL* is expressed only in the pituitary gland **(C1)**, whereas the *PRLΔE1* isoform is found in mutant retinas but also in pituitary gland and in kidney of the normal dog **(C2)**. Amplification of *RPS5* was included as a control **(C3)**.

The canine *PRL* gene is comprised of 5 exons. However, our retinal transcriptomic data^33^ lacks any sequencing reads that align with the first exon of *PRL* (Fig. 1B). Since the first strand for the RNAseq library was synthesized using oligo-dT primers, this may have introduced a 3′ bias in the analysis^34–36^. To rule out 3′ bias, we carried out a simple analysis, calculating the coverage for the first exon, and the coverage of all other exons (except the first), for a small subset of genes (*PRL, MYBPH, CNGA3, MERTK, PDE6H, VCAN, GNGT2, TPH1* and *DES*). This subset included genes with varying transcript lengths and expression levels in the published RNAseq dataset^33^. For each of the genes analyzed, irrespective of the transcript length or expression level, sequence reads from exon 1 were present (coverage > 0), except in the case of *PRL* where the coverage for exon 1 was equal to zero (Table 1). Thus, we inferred that the lack of coverage for *PRL* exon 1 was not due to a 3′ bias in the RNAseq library preparation.

We identified the transcription start sites (TSS) for both pituitary gland- and retina-derived *PRL* by performing a 5′RACE assay. Using an antisense gene-specific primer (GSP) within exon 4 of canine *PRL*, we sequenced and found that the retina-derived transcript has a start site (chr35:20681652; CanFam3.1 assembly) within the exon 2 of *PRL* (Fig. 1C1). For the pituitary gland *PRL,* we identified two distinct TSS; while one transcript starts upstream of exon 1 (Fig. 1C2) as expected (chr35:20683836; CanFam3.1 assembly), a second TSS was identified at the end of the first intron (chr35:20681658; CanFam3.1 assembly) (Fig. 1C3). We refer to this short *PRL* isoform lacking the first exon as *PRLΔE1* in this study.

### The full length *PRL* isoform is not expressed in the dog retina

To further confirm that this retinal isoform lacks exon 1, we tried amplification of full length *PRL* and *PRLΔE1* from two normal dog pituitary glands, and from three normal, three rcd1 and three xlpra2 retinas. PCR amplification using a primer pair spanning the defined translation start and end sites (Fig. 2A1) showed that full length *PRL* is expressed only in the pituitary gland of dogs (Fig. 2B1). However, when the forward primer was designed to span the predicted *(ORFfinder)* first AUG codon for *PRLΔE1* that lies within the second exon (Fig. 2A2), the *PRLΔE1* transcript could be amplified from mutant retinas and pituitary gland, but not from the normal retina (Fig. 2B2). Thus, while the degenerating retinas express the *PRLΔE1* isoform and not the full length *PRL*, the pituitary gland can express both the full length and the short *PRL* isoforms.

Since *PRL* is known to have non-retinal extra-pituitary expression^31^, we examined the expression of the full length *PRL* (Fig. 2C1) and *PRLΔE1* (Fig. 2C2) transcripts by PCR in several other organs and tissues from a normal dog. *PRLΔE1* expression was found only in the kidney of the normal dog but at low expression level. In summary, we confirmed that the diseased retina does not express the full length *PRL* but instead expresses the short *PRLΔE1* isoform.

### *PRLΔE1* expression in degenerating canine retinas is limited to the photoreceptors

**Figure 3:**
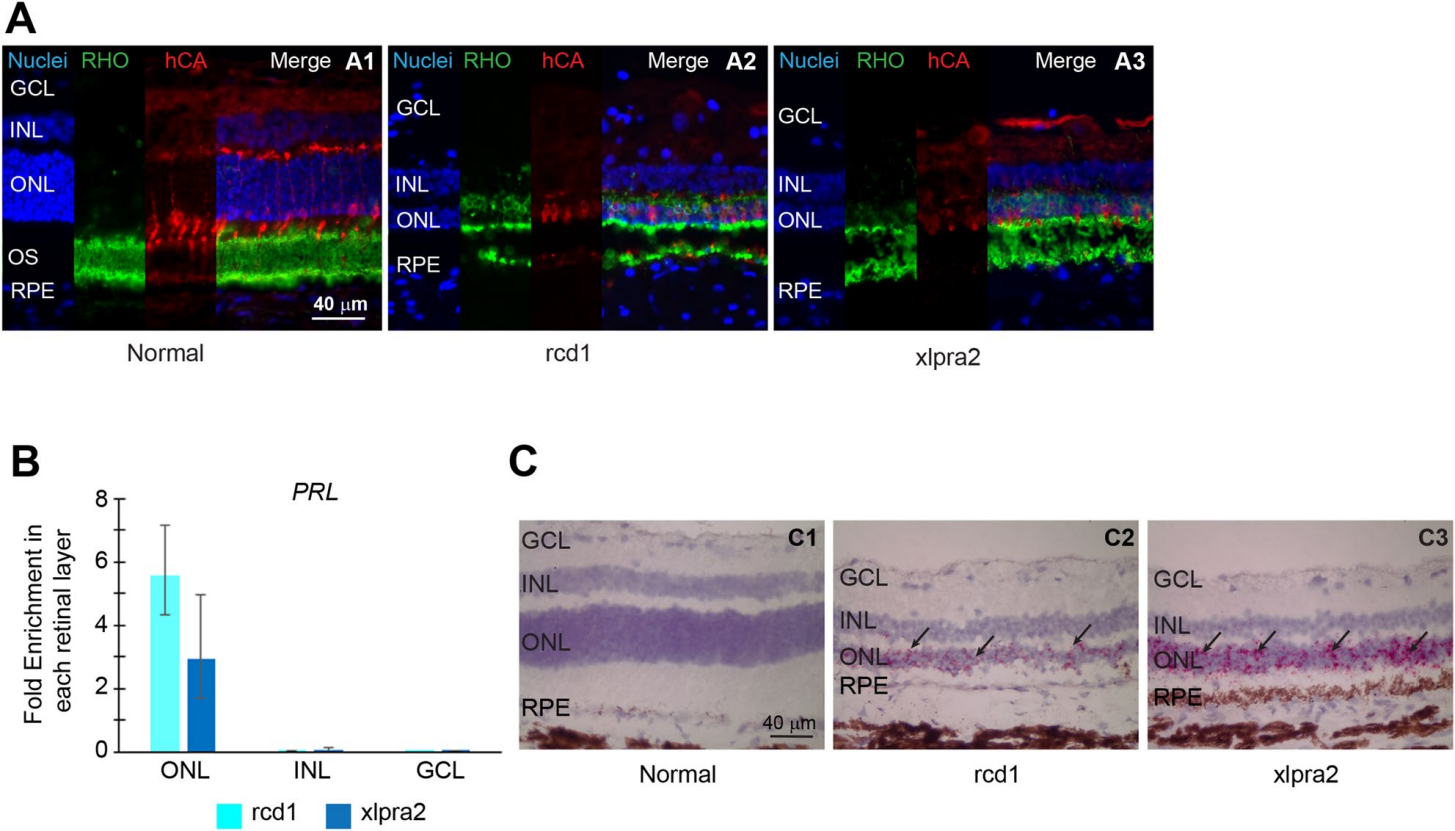
Prolactin expression is upregulated in photoreceptors of mutant canine retinas at advanced stage of degeneration. **(A1-3)** Representative rhodopsin (green)- and cone arrestin (red)-labelled retinal cryosections from normal, rcd1 and xlpra2 dogs showing the extent of ONL loss visualized with nuclear label in degenerating mutant retinas (rcd1: 22 weeks, xlpra2: 41 weeks). (**B)** Fold enrichment of *PRL* mRNA in each retinal cellular layer (ONL, INL, and GCL) in rcd1 and xlpra2 dogs compared to total *PRL* mRNA in all layers taken together (n = 3). **C1-3)** Illustration of RNA-ISH results on retinal cryosections from normal and mutant (rcd1, xlpra2) dogs showing expression of *PRL* restricted to the ONL of mutant retinas.

To identify the specific retinal cellular layers that express the *PRLΔE1* message in normal and mutant dogs, we performed qRT-PCR analysis on RNA obtained by laser capture microscopy (LCM) from each retinal layer and from the entire retinal thickness (Fig. 3A1–3). We examined the enrichment of *PRLΔE1* mRNA expression in each layer by comparing the relative amount of *PRLΔE1* mRNA in each layer to that found in the entire retinal thickness. *PRLΔE1* mRNA was enriched only in the outer nuclear layer (ONL) of the mutant retinas (Fig. 3B). The Ct values obtained for *PRL* in normal retinas were too high (ranging from 37 in some samples to undetermined in others) to unequivocally be distinguished from background, and therefore were not analyzed for layer-specific enrichment.

To confirm that *PRLΔE1* mRNA is expressed only in the ONL, we performed RNA-in situ hybridization (RNA-ISH) on retinal sections from normal and mutant retinas using canine *PRL* specific RNAscope probes targeting nucleotides 7–862 of the full-length mRNA (NM_001008275.2). The *PRLΔE1* mRNA expression was observed specifically in the ONL of the rcd1 and xlpra2 retinas while the normal retina did not show any *PRL* labeling (Fig. 3C1–3). In summary, the results demonstrate that *PRLΔE1* mRNA is upregulated in photoreceptors of rcd1 and xlpra2 retinas at advanced stages of degeneration. Even though our qPCR primers and the RNA-ISH probes do not distinguish full length *PRL* from the *PRLΔE1*isoform, based on our RACE analysis and PCR results, we conclude that the *PRL* message observed in the photoreceptors corresponds to *PRLΔE1*.

### *PRLΔE1* expression in canine photoreceptors is induced shortly after the onset of degeneration

**Figure 4:**
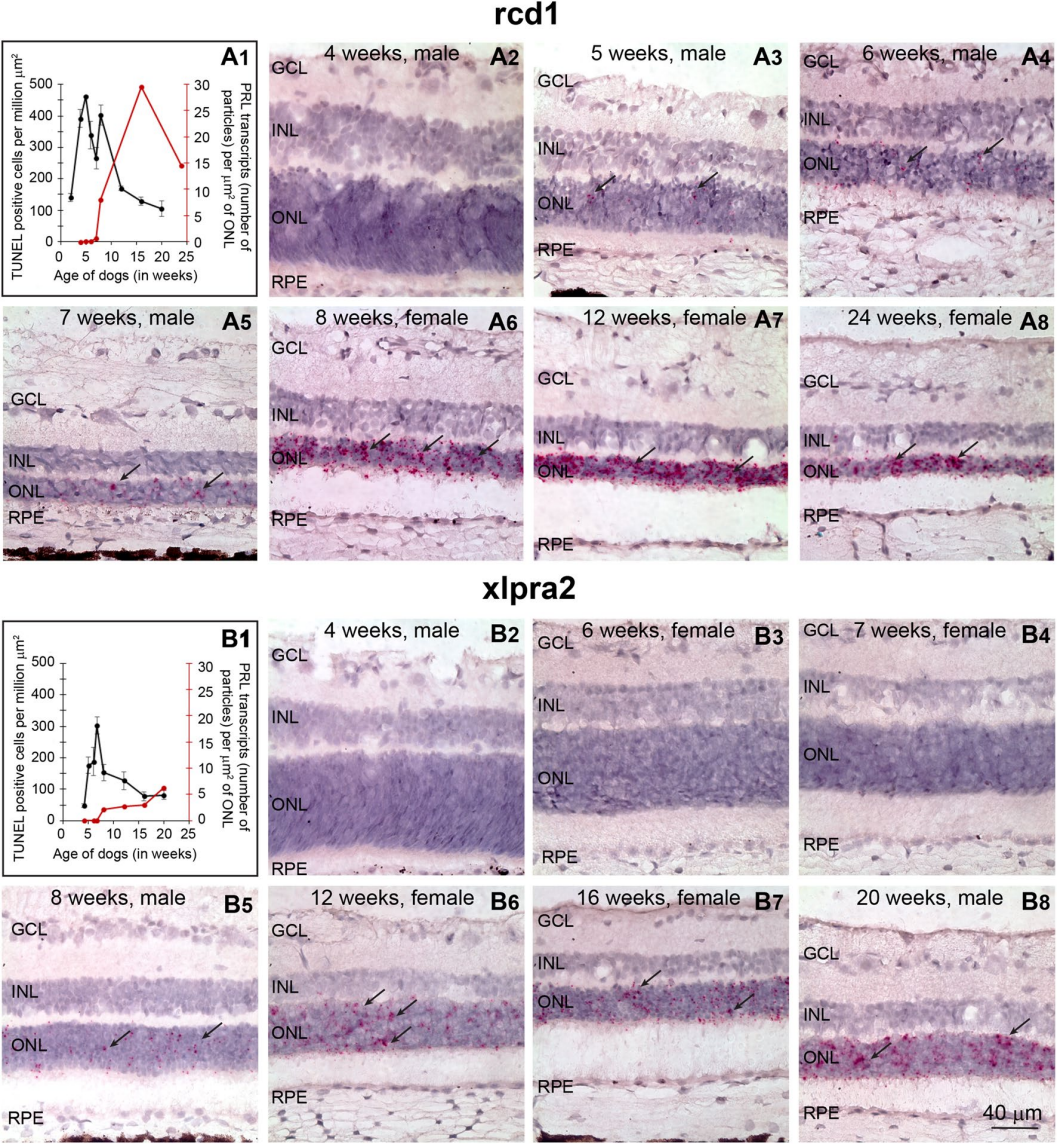
Early onset and increasing expression of *PRL* mRNA with progression of retinal degeneration in dogs. **(A1)** Black line: TUNEL positive photoreceptors measured in the superior/tapetal retina as a function of age in rcd1 dogs. Figure modified from a previous publication^37^ Red line: Quantitation of number of PRL particles detected by RNA-ISH as a function of age in xlpra2 dogs. **(A2–8)** RNA-ISH on cryosections from archival retinal OCT tissue blocks showing expression of PRL mRNA in the ONL of rcd1 dogs at various ages (n = 1 dog per age). **(B1)** Black line: TUNEL positive photoreceptors measured in the superior/tapetal retina as a function of age in xlpra2 dogs. Figure modified from a previous publication^38^. Red line: Quantitation of number of PRL particles detected by RNA-ISH as a function of age in xlpra2 dogs. **(B2–8)** RNA-ISH on cryosections from archival retinal OCT tissue blocks showing expression of PRL mRNA in the ONL of xlpra2 dogs at various ages (n = 1 dog per age). For comparison, the number of TUNEL positive cells per million µm^2^ of ONL ranges from 0–15 in normal dogs^37,38^.

To identify the earliest age at which *PRLΔE1* expression is induced, we performed RNA-ISH on archival retinal tissues collected at various ages from mutant dogs, and correlated expression to the timing of the degenerative process based on cell death kinetics^37,38^ (Fig. 4A1,B1). Note: as stated above, RNA-ISH could not differentiate between full length *PRL* and *PRLΔE1*; however, since *PRLΔE1* was the only isoform identified in retina by PCR, we assume this is the isoform detected by the RNA-ISH probes. In both models, *PRLΔE1* expression was first detected within weeks following the onset of disease. In rcd1 it was first observed at 5 weeks of age which corresponds to the peak of photoreceptor cell death^37^. Its level gradually increased over the course of several weeks and persisted during advanced stages of retinal degeneration (Fig. 4A1–8). In xlpra2 retinas (Fig. 4B) the onset of *PRLΔE1* expression was first observed at 8 weeks of age, which is approximately one week after the peak of cell death in this model^38^, and continued to gradually increase in the ONL as photoreceptor cell death progressed (Fig. 4B1–8). We identified *PRLΔE1* expression in retinas of both male and female dogs. The effect of gender on the levels of expression of *PRLΔE1* is still to be investigated. Expression of *PRLΔE1* mRNA was not detected in retinas of normal dogs at any age (4 to 354 weeks of age) (Supplementary Fig. 1A–G). (RNA-ISH for cone-specific transcript GNGT2 was performed as a control to assess the quality of the archival tissues; Supplementary Fig. 1H).

### Low level expression of PRLΔE1 protein in mutant canine retinas

**Figure 5:**
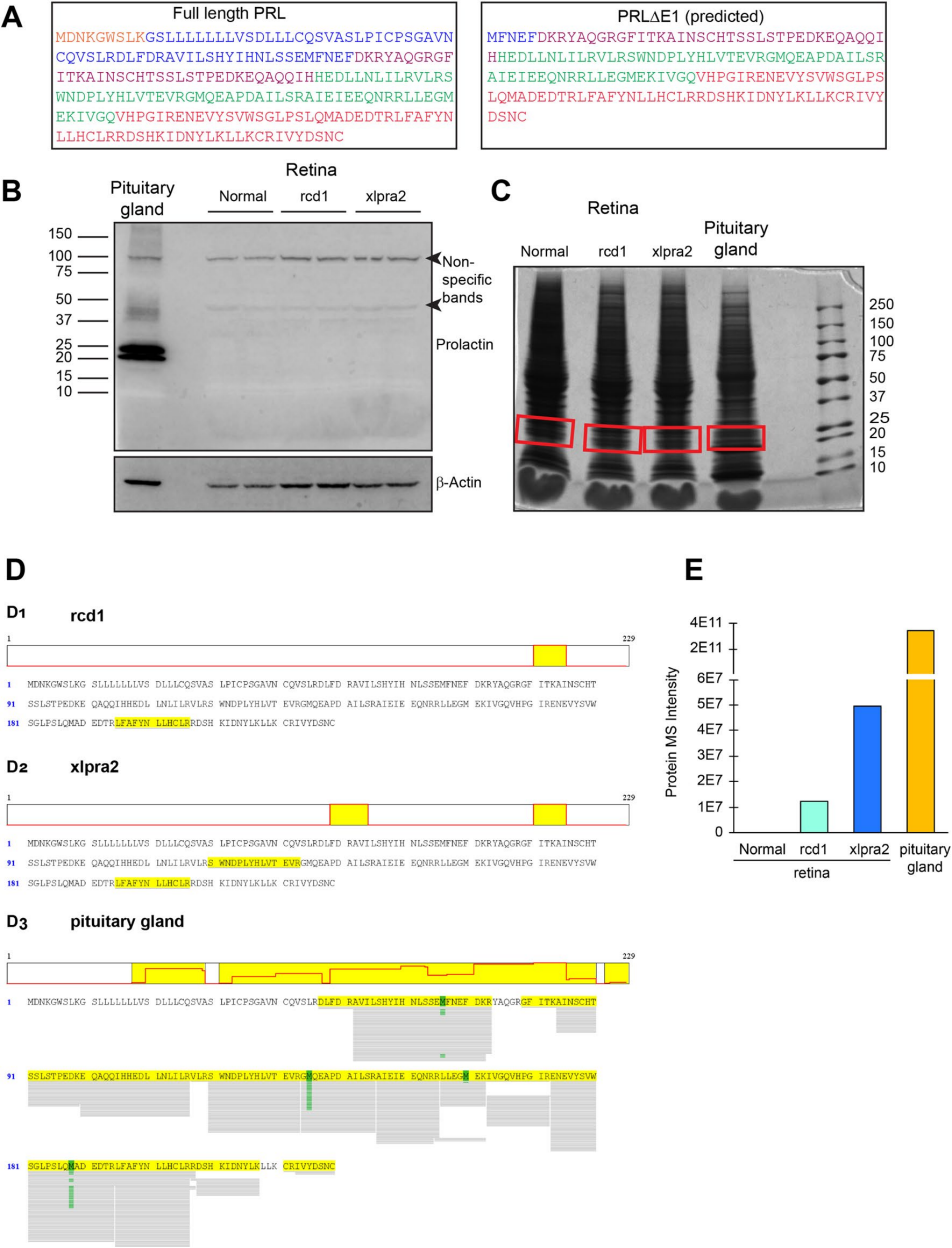
Prolactin protein expression in dog retinas. **(A)** Protein sequence for PRL from dog pituitary gland and predicted protein sequence for PRLΔE1 with the amino acids corresponding to each exon shown with different colors. (**B)** Immunoblot for PRL from dog pituitary gland (n = 1) and normal and mutant retinal extracts (n = 2 for each). (**C)** CBB-stained SDS-PAGE gel for MS/MS analysis with the bands used for analysis highlighted in red. (**D)** Mass spectrometry results showing PRL-specific peptides identified from each sample (highlighted in yellow) **(D1)** rcd1 retina (age: 22 weeks, n = 1), **(D2)** xlpra2 retina (age: 41 weeks, n = 1), and **(D3)** normal canine pituitary gland. (**E)** Protein MS intensity for PRL peptides identified from retinal and pituitary gland samples.

We performed western blot analysis to examine expression of the PRLΔE1 protein in the retinas of normal and mutant (rcd1: 22 weeks and xlpra2: 41 weeks) dogs. The molecular weight of the putative PRLΔE1 protein is expected to be ~ 20 kDa while that of full length PRL is 25 kDa (Fig. 5A). We did not detect any bands of the expected size with a canine-specific polyclonal PRL antibody that should bind to both the full-length and the PRLΔE1 isoforms (Fig. 5B). Thus, liquid chromatography tandem mass spectrometry (LC–MS/MS) was done to identify the proteins in the 20–25 kDa range that were extracted from normal and mutant dog retinas, and from canine pituitary gland (Fig. 5C). While only one peptide corresponding to PRL was detected in the rcd1 sample and two peptides were detected in the xlpra2 sample, PRL peptide coverage for the pituitary gland was expansive (Fig. 5D1–3). LC–MS/MS reanalysis of these samples targeting for specific PRL peptides, identified 2 additional PRL peptides (AIEIEEQNRR and GMQEAPDAILSR) in xlpra2, but no additional PRL peptides were detected for rcd1 and normal retina (data not shown). Thus, very low abundance of prolactin in the mutant retinas was identified, but none was detected in the normal retina (Fig. 5E). The low abundance of PRL peptides in rcd1 and xlpra2 samples did not allow us to confirm the sequence of the specific PRL isoform expressed in the retina as the PRL peptides detected correspond to both the full length PRL protein and the PRLΔE1 isoform.

### The *PRLΔE1* isoform is also expressed in human retinas

**Figure 6:**
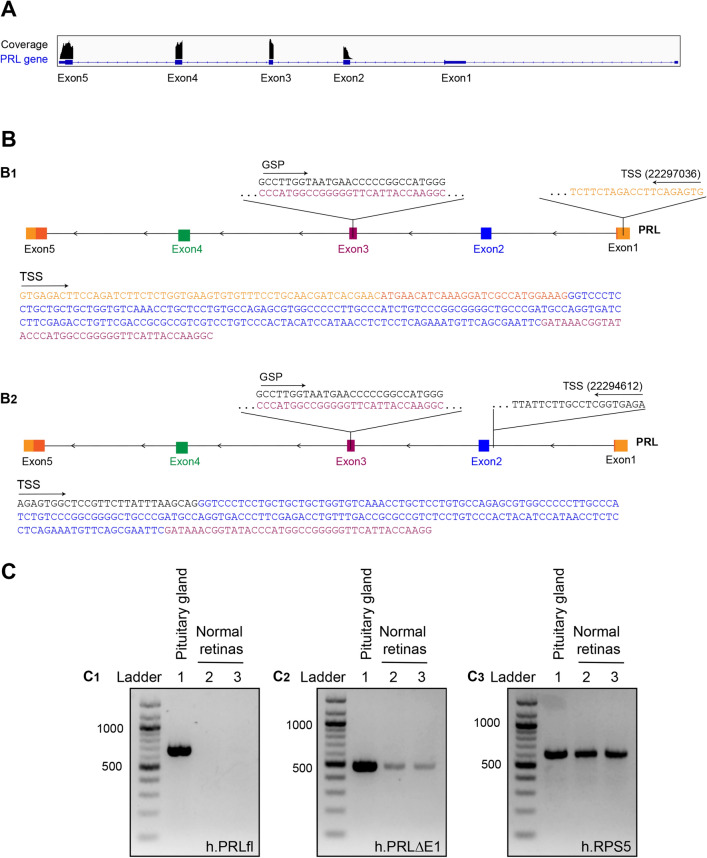
A short Prolactin isoform lacking exon 1 is expressed in the human retina. **(A)** Representative graph derived from Integrated Genomic View (IGV) showing RNAseq (GSE99248)^40^ reads mapped by STAR for *PRL* exons expressed in retinas of normal human eyes. The annotated 6 exons present in the reference are shown, however, only exons 2, 3, 4 and 5 show any coverage. No reads were detected mapping to exon 1a and 1b. (**B)** Transcription start site (TSS) for human pituitary **(B1)** and retinal (n = 3) **(B2)** *PRL* identified by 5′-RACE. GSP = gene-specific primer. **(C)** The full length *PRL (h.PRLfl)* transcript is found in human pituitary gland (n = 1) but not in normal human retinas (n = 2) **(C1)**, whereas the short *PRL* isoform (*h.PRLΔE1*) is expressed in human retinas **(C2)**. Amplification of housekeeping gene *h.RPS5* was included as a control **(C3)**.

In silico analysis of published transcriptomic data from human retina (Retinal transcriptome, Ocular Genomic Institute^39^ and GSE99248^40^) revealed that the sequencing reads for the first *PRL* exon were absent. To experimentally confirm this finding, we extracted RNA from healthy human retinas and compared the 5′RACE sequences for *PRL* in retinas and pituitary gland (Fig. 6A). Whereas the TSS for human pituitary *PRL* sequence extended into the 5′-UTR region upstream of the first exon (chr6:22297036; GRCh38.p13 assembly) (Fig. 6B1), the TSS for human retinal *PRL* included sequences from intron 1 and did not extend into exon 1 and the 5′ UTR (chr6:22294612; GRCh38.p13 assembly) (Fig. 6B2). This was in agreement with the RNAseq data for *PRL* from human retina, and comparable to our findings in dog. Only the *PRLΔE1* isoform, and not the full-length *PRL* transcript, was amplified from human retinas using PCR (Fig. 6C1–3). Thus, our results show expression of a short PRL isoform lacking exon 1 in both canine and human retinas.

### A second PRL isoform lacking exons 1 and 5 is also expressed in canine retinas

We also observed very low-level expression of a second *PRL* transcript in dog retinas. In addition to the absence of the first exon, this isoform also differed in the C-terminal due to altered splicing after exon 4. This *PRL* transcript included the sequence from the fourth *PRL* intron and did not express the fifth exon. This difference in sequence at the C-terminus allowed us to amplify and distinguish this *PRL* isoform (*PRLΔE1E5*) from *PRL* and *PRLΔE1* using a distinct primer for the C-terminal sequence (Supplementary Fig. S2A). *PRLΔE1E5* was amplified from dog pituitary gland cDNA, and normal, rcd1 and xlpra2 retinal cDNAs (Supplementary Fig. S2B). qRT-PCR analysis using primer pairs with the same sense primer and distinct anti-sense C-terminal primers that could distinguish between *PRLΔE1* and *PRLΔE1E5* showed that the *PRLΔE1E5* is expressed in very low amounts, and there is only a 3–7 fold increase in *PRLΔE1E5* expression in mutants compared to normal retinas (Supplementary Fig. S2C). Due to the extremely low abundance of this transcript, we could not identify the alternate *PRL* 3′ end using 3′RACE analysis. An equivalent transcript for human *PRL* was also not identified by PCR. The predicted PRLΔE1E5 protein would have a molecular weight of ~ 13 kDa. However, we did not detect any PRL protein by mass spectrometry in bands excised in the 10–15 kDa range from pituitary gland and normal and mutant retinas suggesting it might not be translated.

## Discussion

Prolactin’s role in light-induced retinal degeneration and in diabetic retinopathy has been investigated for nearly four decades^7,13,20,27,41–43^. The neuroprotective role of prolactin in retina has been attributed to both the full length PRL and the post-translationally cleaved bioactive peptides, called vasoinhibins, which enter the retina either through circulation, or are produced endogenously. In our previous work^33^ focused on understanding common pro-survival/apoptotic pathways in two canine models at advanced stages of retinal degeneration, we identified PRL as one of the highest expressed transcripts. This study now confirms and further extends these findings by identifying novel short *PRL* isoforms expressed in the retinas of two naturally occurring canine models of inherited retinal degeneration, one of which is expressed also in humans.

Most mammalian vertebrates, including dogs and humans, have a single prolactin gene comprised of 5 exons and 4 introns^44,45^. Through gene duplication events in rodents, the *PRL* gene family has expanded to form a single locus containing multiple *PRL*-like genes, each with distinct expression pattern and function^46,47^. A second brain and retina-specific *PRL* gene has been identified in some non-mammalian vertebrates that is expressed in all three retinal cellular layers and involved in retinal development in fish^48^. There are sporadic reports of existence of transcriptional variants of extra-pituitary *PRL* in mammals, including humans^49–51^; however, no retinal transcriptional variants have been described to this date in mammals.

In this study, we identified in dog retinas the expression of two *PRL* isoforms distinct from the full length *PRL* expressed in pituitary glands. The major *PRL* isoform in dog retinas lacks the first exon (*PRLΔE1*) and its expression was highly upregulated in degenerating retinas. This isoform closely resembled the canine Ensembl transcript *PRL*-201 (ENSCAFT00000049544.2- Ensembl release 99) that also lacks the first exon and has a TSS within the first intron. A second retinal *PRL* transcript (*PRLΔE1E5*) expressed in normal retinas was identified and was upregulated to a much lower extent compared to *PRLΔE1* in degenerating retinas. *PRLΔE1E5* terminated within the fourth *PRL* intron and thus, lacked exon 5 in addition to the exon 1. This transcript resembled the archival canine Ensembl transcript (ENSCAFT00000049544.1; Ensembl release 97).

Expression of *PRL* mRNA lacking the first exon that is comparable to the *PRLΔE1* mRNA from dogs was also detected in healthy human retinas. Prolactin is expressed in all retinal cellular layers and in the RPE of normal rats and mice^10,15,32^. However, unlike human retinas, *PRLΔE1* expression was undetectable in normal adult dogs and was observed only in the photoreceptors of retinas undergoing degeneration. The earliest expression of *PRLΔE1* was detected at or immediately after the peak of photoreceptor cell death in the rcd1 and xlpra2 models of retinal degeneration. Thus, *PRLΔE1* expression in the retina is likely induced by the onset of photoreceptor loss and maintained as a result of ongoing degeneration.

A number of factors have been implicated in direct or indirect control of extrapituitary PRL expression including hormones (estrogen, progesterone, endothelin-1, adrenergic hormones), cytokines (such as TNFα, IL1α and β), growth factors (EGF, PDGF) and calcium/calmodulin^30^. Although many of these factors are altered during retinal degeneration^33,37^, it is currently unknown whether any of these factors are involved in inducing *PRLΔE1* expression following photoreceptor cell death. Investigations into alternative promoters as well as potential factors involved in *PRLΔE1* transcription are ongoing. Serum PRL levels differ between males and females as a result of differential mRNA expression and different stimuli that control secretion of PRL from the lactotrophs of pituitary gland^52^. Gender-specific differences in extra-pituitary PRL expression has been reported in the CNS^53^ and cochlea^54^ in rodents ; however, the mechanism governing these is not defined. In this study, we have qualitatively identified *PRLΔE1* expression in both male and female mutant dogs but quantitative analysis was not conducted. Since this isoform is distinct from that expressed in the pituitary gland, it would be of interest to evaluate in future studies if any gender differences influence the expression of *PRLΔE1*.

PRL has been shown to have a neurogenic and neuroprotective role in the CNS^55^. Studies in rats and mice have primarily attributed a pro-survival role to PRL in retina. In rats, increasing the circulating levels of PRL by inducing hyperprolactinemia was shown to protect the retina in a light induced retinal damage model by limiting retinal gliosis and modulating the expression of a number of neurotrophins^13^. PRL was also shown to protect rat and mouse RPE from oxidative damage by decreasing sirtuin-2 expression and inhibition of TRPM2 channels^15^. Further studies will be required to address whether PRLΔE1 has a similar pro-survival role in canine retinas undergoing degeneration.

The dog PRL protein sequence is 80% identical to the human PRL. The structure of human PRL protein and the residues essential for its interaction with the PRL receptor (PRLR) homodimer have been identified. PRL forms a 4 helix bundle^56^ that binds to its receptors via two distinct epitopes, site 1 and site 2^22,57,58^. Almost all of the sites 1 and 2 amino acid residues are conserved in dog PRL. In the present study, we detected prolactin protein in rcd1 and xlpra2 retinal extracts, but not in the retina of a normal dog. Due to very small amounts of PRL protein in the retina, only a few peptides could be detected by mass spectrometry, thus limiting our efforts to determine the sequence of specific retinal PRL isoforms. However, based on the AUG codon availability, the protein product of the *PRLΔE1* mRNA could be predicted to initiate at methionine-36 of the full length PRL protein. (The first 28–30 amino acids of PRL pro-hormone comprising the secretion signal peptide are cleaved in the final PRL protein; the full length PRL hormone thus starts with a leucine in the first position). This altered start position would result in loss of the secretion signal peptide, the N-terminal loop, and the first helix, but still preserve all of the amino acids in site 1 and site 2 of PRL. Based on SecretomeP^59^ prediction, PRLΔE1 has high probability (NM-score 0.740) of being secreted via the unconventional protein secretion pathways^60^. The N-terminal loop moderately affects the binding affinity of the full length PRL to its receptor^61^. Thus, one could speculate if PRLΔE1 folds correctly to form the remaining helix bundles, then it may be able to bind PRLR and activate the JAK-STAT pathway^22^. Additionally, PRLΔE1 retains many of the potential cathepsin-D sites that are required to generate vasoinhibin fragments^7^. However, the first helix that has been shown to be required for the anti-angiogenic activity of vasoinhibins^62^ is lacking in this isoform. Therefore, it is unlikely that the PRLΔE1 isoform generates bioactive vasoinhibin peptides. Further studies will be directed at identifying the function of this PRL isoform in retinal degeneration.

The amount of the second *PRL* mRNA isoform (*PRLΔE1E5*) in this study was extremely low compared to *PRLΔE1* mRNA and we did not detect the putative PRLΔE1E5 protein by mass spectrometry among the proteins isolated from the ~ 10–15 kDa region of the gel. Therefore, the *PRLΔE1E5* may not be translated into protein.

In summary, we present evidence for expression of novel PRL isoforms in the retinas of dogs affected with two non-allelic inherited retinal degenerative diseases. In the retina, the expression of this PRL isoform lacking the first exon is limited to the photoreceptors, the primary retinal cells affected in both diseases. The onset of expression of this isoform in dog retina is correlated with the early peak of cell death, thus underlining its role in retinal degeneration. Future studies are needed to determine whether this PRL isoform has a pro-survival role in the retina or contributes to the degenerative process. Furthermore, we have shown that a similar *PRL* mRNA lacking exon 1 is also expressed in human retina. Unlike in man and dog, the existence of multiple PRL-like genes in the mouse^46^ complicates analysis of the function of PRL in the retina. This further emphasizes the value of the dog as a model system to explore the role of retinal PRL isoforms in disease. In addition, the sustained expression of PRL throughout the course of retinal degeneration could provide a large therapeutic window aimed at modulating its function.

## Methods

### Animals use statement

Neuroretinas from six normal dogs (age: 24–26 weeks), five affected homozygous rcd1/*PDE6B* mutant dogs (age: 22 weeks) and five affected homozygous xlpra2/*RPGR* mutant dogs (age: 41 weeks) were used for this study. Additionally, pituitary glands and other tissues were obtained from 3 normal dogs for use in this study. The ages selected for the mutant animals corresponded to an age when the ONL thickness in the retina had decreased to less than 50% of that of normal dogs^37,38,63^. All dogs were housed under identical conditions (diet, ambient illumination with cyclic 12 h ON-12 h OFF light) at the Retinal Disease Studies (RDS) facility of the University of Pennsylvania. The studies strictly adhered to the ARVO Statement for the Use of Animals in Ophthalmic and Vision Research and were approved by the Institutional Animal Care and Use Committee (IACUC) of the University of Pennsylvania. For tissue collection, dogs were euthanized with an intravenous injection of sodium pentobarbital. After enucleation, the neuroretina was removed from the RPE/choroid, and both tissues separately stored at − 80 °C until further experiments, or the posterior eyecup dissected into four quadrants and embedded in Optimal Cutting Temperature (OCT) medium without prior fixation. In addition, several archival OCT-embedded paraformaldehyde (PFA)-fixed retinas were used for in situ hybridization. Retinal fixation and embedding protocol was designed to minimize RNase activity. All dogs used in this study are listed in Supplementary Table 1A and 1B.

### Postmortem human eye collection, isolation of retina, and AMD grading

Human eyes were collected at the Lions Eye Institute for Transplant and Research (Tampa, Florida, USA) from non-diabetic Caucasian donors with a death-to-preservation interval of less than 5 h. Informed consent was obtained in compliance with the Florida Agency for Healthcare Administration (AHCA) statute 59A-1 and the Eye Banking Association of America guidelines. All experiments with human tissues were conducted in accordance with the university safety guidelines and regulations. All experimental protocols involving the use of postmortem human eyes were exempted of ethical approval by Institutional Review Boards at both the Mayo Clinic at Rochester and the University of Pennsylvania. Immediately after enucleation, anterior segment was removed by making a circumferentially cut 5–6 mm away from the limbus. The posterior eye globe was placed under a dissecting stereomicroscope. Using a 1000 ± 2.5 µm ruby bead placed over the optic nerve as a standard size reference, digital images were captured under direct light illumination at 1.5X and 3X magnifications. The neuroretina was gently separated from the RPE-choroid, excised into smaller pieces in 1X PBS and stored in cryovials in RNAlater solution. Additional images of the posterior eye cup with the RPE-choroid were obtained.

To identify healthy retinas, the images were graded using the Minnesota Grading System (MGS), following the Age-Related Eye Disease Study (AREDS) criteria to determine the level of AMD^64,65^. Punches were obtained from peripheral areas of retinas of eyes with no drusen or a few small drusen < 63 μm that were categorized as normal (Grade 1). The age, sex and location of the punches from the 2 normal retinas used in this study are detailed in Supplementary Table 2.

### RNAseq mapping and evaluation of exon coverage and 3′ bias

Single-end canine RNAseq reads from our transcriptomic study comparing normal retinas to rcd1 and xlpra2 retinas^33^ [European Nucleotide Archive (ENA) study accession number PRJNA382537] were mapped to the CanFam3.1 dog reference genome. The human retinal RNAseq fastq files (30 samples), obtained from a study published by Kim et al. (ENA study accession number PRJNA387704)^40^, were mapped to human reference genome GRCh38. Reads were aligned using STAR, version 2.5.2a, with default settings^66^. The genome indices for both references were prepared as indicated in the manual. The mapped .sam file generated by STAR was then converted to .bam. Using samtools version 1.1, the reads were sorted by chromosome, and finally the sorted file was indexed^67^.

Exon coverage and 3′ bias in the canine retinal transcriptomic dataset^33^ was evaluated using the “depth” tool from Samtools. RNAseq data from only the 3 rcd1 and 3 xlpra2 samples was used. Several genes were selected from the dataset to represent a range of transcript lengths (*PRL, MYBPH, CNGA3, MERTK, PDE6H, VCAN, GNGT2, TPH1* and *DES*). Two .bed files were prepared for each of the genes. The first one encompassed the positions of all the exons but excluded exon 1; the second .bed file contained only the interval encompassing exon 1. Using the .bed files as input, the Samtools “coverage” option was then used to evaluate the coverage per base for exon 1 of each of the selected genes and for the remaining exons. The output from Samtools was piped into an awk script to select the coverage value, all the coverage values for each line (and therefore for each position up to the whole interval) were summed, and the average of the coverage and the standard deviations were calculated.

### RNA isolation and cDNA preparation

All tissues (neuroretina, retinal pigment epithelium-choroid, pituitary gland, visual cortex, cerebellum, breast, ovary, lymph node, spleen tonsil, skin, lung, liver and kidney) collected from dogs after euthanasia were homogenized in TRIzol (Invitrogen, ThermoFisher Scientific, Waltham, MA, USA) using the BeadBug microtube homogenizer (Benchmark Scientific, Edison, NJ, USA) and total RNA was extracted using the standard Trizol protocol. RNA was treated with Turbo DNA-free kit (ThermoFisher Scientific) to remove genomic DNA contamination. RNA concentrations and quality were assessed on Nanodrop One (ThermoFisher Scientific).

RNA from 2 normal human neuroretinal samples was isolated using the Qiagen RNeasy kit (Qiagen, ThermoFisher Scientific) following the manufacturer’s protocol. Human pituitary mRNA was purchased from Takara Biosciences (Mountain View, CA, USA). Total RNA and mRNA was reverse transcribed into cDNA using the High Capacity RNA to cDNA kit (Applied Biosystems, ThermoFisher Scientific).

### Laser capture microscopy and anti-sense RNA amplification

Laser Capture Microdissection (LCM) was used to isolate the three cellular layers (outer nuclear layer and inner segments, ONL; inner nuclear layer, INL; ganglion cell layer, GCL) from unfixed, OCT-embedded retinas of three normal, three rcd1 and three xlpra2 affected dogs. LCM was also applied to collect full retinal sections that included all three layers. Briefly, 12-micron thick sections located within the superior-temporal retinal quadrant were collected on RNase-free PEN-membrane slides (Zeiss, Gottingen, Germany). The sections were stained with hematoxylin for 2 min and dehydrated by quick passage through increasing concentrations of ethanol. Microdissection was carried out immediately using a Leica LMD 7000 microscope. Each of the three retinal layers and the full retina section were dissected and collected individually in AdhesiveCap clear tubes (Zeiss). The tubes were stored in dry ice immediately after collection.

RNA was purified from LCM sections on the same day using RNeasy Plus Micro Kit (Qiagen, ThermoFisher Scientific). First strand cDNA was synthesized from RNA using SuperScript III (ThermoFisher Scientific). A single round of antisense RNA amplification was performed using the MEGAScript T7 Transcription kit (ThermoFisher Scientific). Purity of each cellular layer was verified by qPCR analysis of layer-specific genes [Neural Retina Leucine Zipper (NRL) for ONL; Calcium Binding Protein 5 (CABP5) for INL; Synuclein Gamma (SNCG) for GCL].

### Conventional PCR analysis

Expression of *PRL* transcripts was analyzed in tissues (listed above) and in retinal samples by PCR analysis. *RPS5* was used as an endogenous control gene (Fig. 2B3,C3)^68^. All primers used for PCR were designed manually (listed in Supplementary Table 3). PCR was performed on an Applied Biosystems ProFlex Base thermocycler (ThermoFisher Scientific) using Phusion polymerase enzyme (New England Biolabs Inc., Ipswich, MA, USA). PCR products were visualized on 2% TAE agarose gels stained with ethidium bromide.

### Quantitative real time PCR (qRT-PCR) analysis

Primers used for qRT-PCR analysis were designed using the PrimerQuest Tool from Integrated DNA Technologies (IDT, Inc., Coralville, IA, USA) (listed in Supplementary Table 3). qRT-PCR was performed on a ViiA 7 Real-Time PCR System (348-well format) (Applied Biosystems, ThermoFisher Scientific). Each 20 µL reaction contained 10 µL of SYBR Green PCR Mastermix (Life Technologies, ThermoFisher Scientific), 250 nM each of forward and reverse primers and either 5 ng (whole retina) or 100 ng (LCM samples) of cDNA. Each sample was analyzed in triplicates. Comparative deltaCt method was used for relative comparison of gene expression levels using GAPDH as an endogenous control for normalization. Fold change was calculated as 2^-(ΔΔCt)^ and statistical significance was calculated by an unpaired homoscedastic t-test using a two-tailed distribution.

### 5′-rapid amplification of cDNA ends (5′-RACE) analysis

Transcription start site for retinal *PRL* transcript was identified using 5′-RACE analysis. SMARTer RACE kit (Takara Bio USA Inc, Mountain View, CA, USA) was used for 5′-RACE as per manufacturer’s guidelines. *PRL* gene-specific primers (Supplementary Table 3) were also designed following guidelines in the manual. Briefly, first strand RACE ready cDNA was synthesized from retinal RNA. 5′-RACE products were amplified from the cDNA using 5′ RACE CDS Primer A and gene-specific primers, amplicons were purified using the Nucleospin Gel and PCR Clean up kit and cloned into linearized pRACE plasmid. After transformation and culturing on LB agar-ampicillin plates, plasmids were isolated from bacterial colonies and sequenced using M13Forward and M13Reverse primers. Sequences were aligned to canine (Broad CanFam3.1/canFam3) or human (GRCh38.p13) genomes using the Blat tool from UCSC Genome browser and mapped to the *PRL* gene using the NCBI Genome Data Viewer.

### RNA-in situ hybridization

Localization of the site of *PRL* mRNA expression in the retina was visualized by RNA-in situ hybridization (RNA-ISH) using the RNAscope assay [Advanced Cell Diagnostics (ACDBio), BioTechne, Newark, CA, USA]. Canine *PRL*-specific RNAscope probes spanning nucleotides 7 to 862 (NM_001008275.2) were designed by ACDBio (catalog no. 535781). Ten micron-thick frozen sections were cut from both unfixed and PFA-fixed OCT-embedded retinas from normal and mutant dogs. Sections from unfixed retinas were post-fixed in 4% PFA for an hour at 4 °C before proceeding with the pretreatment. Target retrieval was performed on fixed frozen sections by heating the slides in Target Retrieval buffer at 88 °C for 10 min followed by protease digestion. RNA-ISH was performed using the RNAscope 2.5 HD Assay-Red following the guidelines in the product manual. After hematoxylin staining, the retinal sections were examined and images were captured using a bright field microscope (Axioplan, Carl Zeiss Meditec GmbH, Oberkochen, Germany) with a 40× objective. For quantification, complete sections were scanned using Aperio Digital Pathology Slide Scanner (Leica Biosystems, Buffalo Gove, IL, USA) and analyzed using the open-source Java image processing program Fiji (ImageJ)^69^. PRL transcripts stained in red were counted only in the ONL and normalized to the area of the ONL in each retinal section.

### Immunohistochemistry

Retinal integrity and degeneration in mutant retinas was visualized by immunohistochemistry (IHC) using photoreceptor-specific antibodies directed against rod opsin (MAB5316; 1:200 dilution; EMD Millipore, Billerica, MA, USA), and goat anti-human cone arrestin (W. Beltran, Univ. of Pennsylvania; 1:100) on 10 µm-thick retinal sections as previously described^33^. Briefly, the sections were incubated overnight with the primary antibodies at 4 °C after a blocking step with buffer containing 5% BSA and 4.5% fish gelatin in PBST. Antigen antibody complexes were visualized with Alexa-fluor labeled secondary antibodies (Invitrogen, ThermoFisher Scientific, 1:200). Hoechst 33342 nuclear stain (Thermo Fisher Scientific) was used to label cell nuclei. Slides were mounted in Gelvatol mounting medium (containing polyvinyl alcohol and glycerol), examined with an epifluorescence microscope (Axioplan, Carl Zeiss Meditec), and images were digitally captured using Spot 4.0 camera, and processed using Adobe Photoshop and Illustrator programs for display.

### Western blot

Western blot assay was performed to detect full length and shorter PRL proteins in retinal samples. Neuroretinal extracts were prepared from 2 normal, 2 rcd1 and 2 xlpra2 retinas as previously described^33^. Briefly, retinas were homogenized using a bead homogenizer in a buffer containing 0.23 M sucrose, 2 mM EDTA, 5 mM Tris HCl, pH 7.4, 1% Tx100 and a protease and phosphatase inhibitor cocktail (Halt, ThermoFisher Scientific) and then sonicated. The samples were centrifuged and total protein concentration in the supernatant measured by BCA assay. 50 µg of total protein from each sample was resolved on an 4–20% Tris Glycine gel (Invitrogen, ThermoFisher Scientific), transferred to a PVDF membrane for 5 min (iBLOT, Invitrogen, ThermoFisher Scientific) and immunoblotted using an antibody (1:1000 dilution) generated specifically to recognize the canine PRL (GenScript, Piscataway, NJ). After incubation with infrared dye-tagged secondary antibodies (IR-dye 800CW, Odyssey Fc, Licor, Lincoln, NE), protein bands were visualized on a digital imaging system. The same blot was also incubated with an antibody against b-Actin (1:3000, ab8226, Abcam, Cambridge, MA) and detected with an IRDye 680RD secondary antibody (Odyssey Fc, Licor). A Note: A polyclonal human prolactin antibody from R and D Systems (catalog no. AF682) targeting residues 29–227 of human prolactin was also tested (1:500 dilution) but did not recognize canine prolactin.

### LC–MS/MS analyses and data processing

Liquid chromatography tandem mass spectrometry (LC–MS/MS) analysis was performed as previously described^70^ by the Proteomics and Metabolomics Facility at the Wistar Institute (Philadelphia, PA) using a Q Exactive Plus mass spectrometer (ThermoFisher Scientific) coupled with a Nano-ACQUITY UPLC system (Waters Corporation, Milford, MA, USA). The gel regions (20 kDa to 25 kDa) were excised, digested in-gel with trypsin and injected onto a UPLC Symmetry trap column (180 μm i.d. × 2 cm packed with 5 μm C18 resin; Waters Corporation). Tryptic peptides were separated by reversed phase HPLC on a BEH C18 nanocapillary analytical column (75 μm i.d. × 25 cm, 1.7 μm particle size; Waters) using a 95 min gradient formed by solvent A (0.1% formic acid in water) and solvent B (0.1% formic acid in acetonitrile). A 30-min blank gradient was run between sample injections to minimize carryover. Eluted peptides were analyzed by the mass spectrometer set to repetitively scan m/z from 400 to 2000 in positive ion mode. The full MS scan was collected at 70,000 resolution followed by data-dependent MS/MS scans at 17,500 resolution on the 20 most abundant ions exceeding a minimum threshold of 20,000. Peptide match was set as preferred; exclude isotopes option and charge-state screening were enabled to reject unassigned and single charged ions.

Peptide sequences were identified using MaxQuant 1.6.8.0^71^. MS/MS spectra were searched against a UniProt dog protein database (October 2019) and a common contaminants database using full tryptic specificity with up to two missed cleavages, static carboxamidomethylation of cysteine, variable oxidation of methionine, and protein N-terminal acetylation. Consensus identification lists were generated with false discovery rates set at 1% for protein and peptide identifications.

**Table 1:** Average coverage and standard deviations of the first exon (A), and for the remaining exons (B) of the transcripts for MYBPH, CNGA3, MERTK, PDE6H, VCAN, GNGT2, TPH1, DES, and PRL genes, for each of the six samples analyzed (IDs reported).

	MYBPH	CNGA3	MERTK	PDE6H	VCAN	GNGT2	TPH1	DES	PRL
Length (basepairs)	3552	3512	3074	849	8829	1624	4073	1625	786
**(A) Exon1**									
SRR5443382	7.09	7.72	2.46	736.6	7.61	198.22	250.67	45.65	0
	(1.75)	(0.78)	(0.67)	(152.8)	(2.07)	(76.47)	(50.42)	(19.46)	(NA)
SRR5443383	4.6	4.6	3.37	765	8.98	196.12	320.09	52.1	0
	(2.57)	(2.57)	(1.06)	(183.6)	(1.62)	(83.18)	(79.03)	(20.71)	(NA)
SRR5443384	9.89	7.89	5.2	861.3	8.94	278.06	326.86	46.32	0
	(5.91)	(2.36)	(0.44)	(181.3)	(0.96)	(121.63)	(86.31)	(13.97)	(NA)
SRR5443385	6.83	6.84	1.86	641.6	11.8	192.48	254.83	82.49	0
	(2.44)	(2.44)	(0.78)	(131.5)	(1.6)	(65.44)	(70.72)	(34.98)	(NA)
SRR5443386	9	9	1.76	1072.5	9.69	252.53	262	81.4	0
	(4.7)	(4.7)	(0.57)	(131.5)	(4.55)	(101.85)	(54.53)	(35.99)	(NA)
SRR5443387	8.64	8.64	1	656.37	9.98	168.18	187.64	49.15	0
	(2.96)	(2.95)	(0)	(160.37)	(1.42)	(73.14)	(54.58)	(21.42)	(NA)
**(B) Rest of the exons**									
SRR5443382	12.3	12.88	9.77	1195	30.39	293.72	246.66	236.04	38.54
	(5.99)	(4.92)	(3.66)	(621.5)	(11.92)	(95.74)	(78.06)	(71.79)	(13.89)
SRR5443383	7.891	9.88	9.6	1362.05	36.49	357.62	310.76	188.34	31.41
	(2.36)	(5.91)	(3.2)	(689.44)	(14.8)	(98.47)	(95.77)	(54.62)	(12.37)
SRR5443384	14.56	14.56	10.8	1398.7	33.27	412.79	346.31	128.01	23.32
	(7.66)	(7.66)	(3.45)	(718.4)	(13.2)	(115.91)	(101.52)	(34.53)	(9.4)
SRR5443385	8.9	8.9	8.09	1014.2	42.33	299.57	261.87	301.81	60.29
	(5.16)	(5.16)	(2.9)	(526.4)	(15.7)	(75.85)	(74.64)	(92.11)	(21.09)
SRR5443386	10.45	10.46	8	1462.33	45.39	411.62	296.65	325.05	118.57
	(7.3)	(7.3)	(3.27)	(759.4)	(16.03)	(104.56)	(74.68)	(126.91)	(38.66)
SRR5443387	11.2	11.2	7.12	1181.03	41.66	317.82	207.83	251.5	77
	(6.95)	(6.95)	(3.28)	(637.99)	(41.66)	(89.81)	(61.71)	(87.6)	(22.36)
**(C) Exon1 average**	7.68	7.45	2.61	788.90	9.50	214.27	267.02	59.52	0.00
	(3.68)*	(2.87)*	(0.67)*	(158.23)*	(2.35)*	(89.04)*	(67.32)*	(25.76)*	(NA)*
Rest of the exons average	10.88	11.31	8.90	1268.89	38.26	348.86	278.35	238.46	58.19
	(6.17)*	(6.40)*	(8.90)*	(663.14)*	(14.49)*	(97.51)*	(82.19)*	(83.26)*	(21.93)*

Standard deviations are shown for each average. For all transcripts except PRL, whether the average coverage is the order of the tens or in the order of hundreds for the remaining transcripts, a coverage greater than zero is detectable for exon 1. Samples with an average coverage smaller than PRL by an order of magnitude still show a detectable exon 1. In (C), averages for the six samples are shown. Values in parentheses are standard deviations.

*Indicates actual square root of the average of the square of the standard deviations.

## Supplementary Information

Supplementary Information.
