# Supplementary material for: Short prolactin isoforms are expressed in photoreceptors of canine retinas undergoing retinal degeneration
Source: Sci Rep. 2021 Jan 11;11:460. doi: 10.1038/s41598-020-80691-6 (PMC7801730; doi:10.1038/s41598-020-80691-6)
Supplement: Supplementary file 1 — Supplementary Information. [file 41598_2020_80691_MOESM1_ESM.docx]

**Supplementary Information**

**Short Prolactin isoforms are expressed in photoreceptors of canine retinas undergoing retinal degeneration.**

Raghavi Sudharsan^1*^, Leonardo Murgiano^1^, Hsin-Yao Tang^2^, Timothy W. Olsen^3^, Venkata R. M. Chavali^4^, Gustavo D. Aguirre^1^, William A. Beltran^1*^

1 Division of Experimental Retinal Therapies, Department of Clinical Sciences & Advanced Medicine, School of Veterinary Medicine, University of Pennsylvania, Philadelphia, PA 19104, USA.

2 Proteomics and Metabolomics Facility, The Wistar Institute, Philadelphia, PA 19104, USA.

3 Department of Ophthalmology, Mayo Clinic, Rochester, MN 55905, USA.

4 Department of Ophthalmology, Perelman School of Medicine, University of Pennsylvania, Philadelphia, PA 19104, USA.


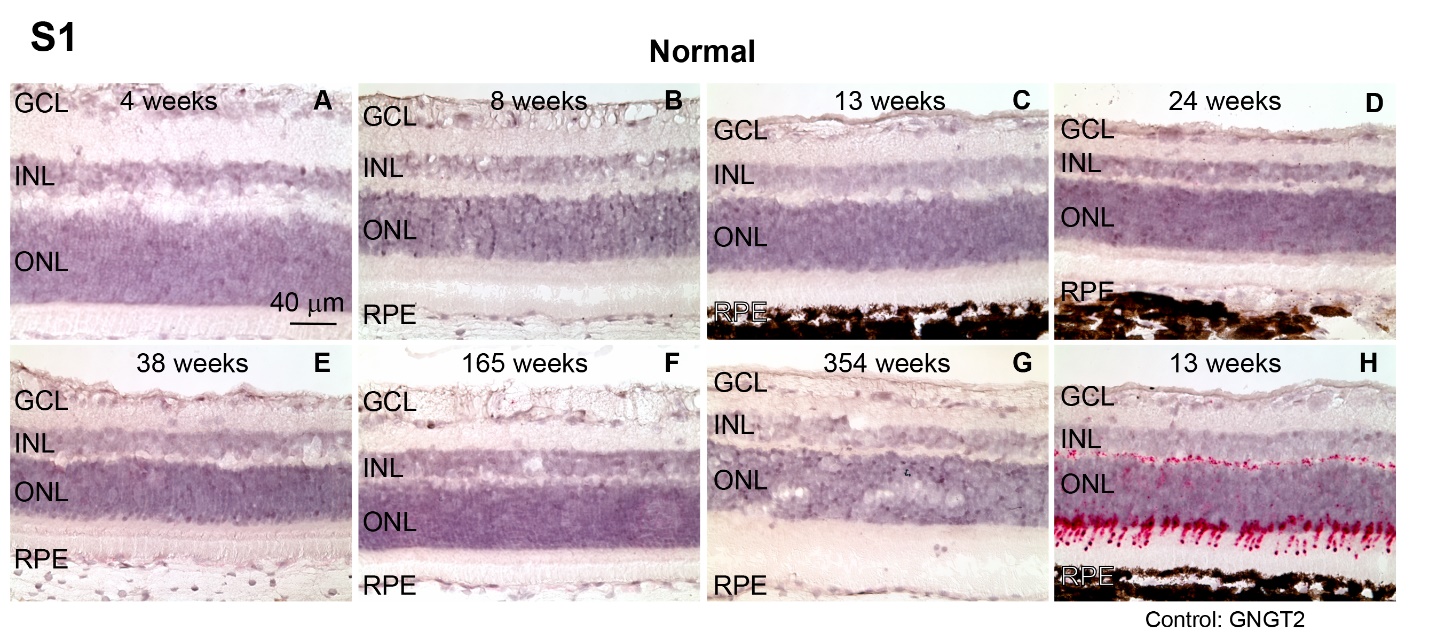


**Supplementary Figure S1:** RNA-ISH on archival retinal cryosections showing lack of expression of *PRL* in the ONL of normal dogs **(A-G)** at various ages (n=1 dog at each age). *GNGT2* RNA-ISH labeling was performed on another retinal cryosection for each dog as a positive control for the technique. A representative figure is shown in **H**, and shows distinct expression pattern of the cone-specific gene *GNGT2* in the cone photoreceptor cells.


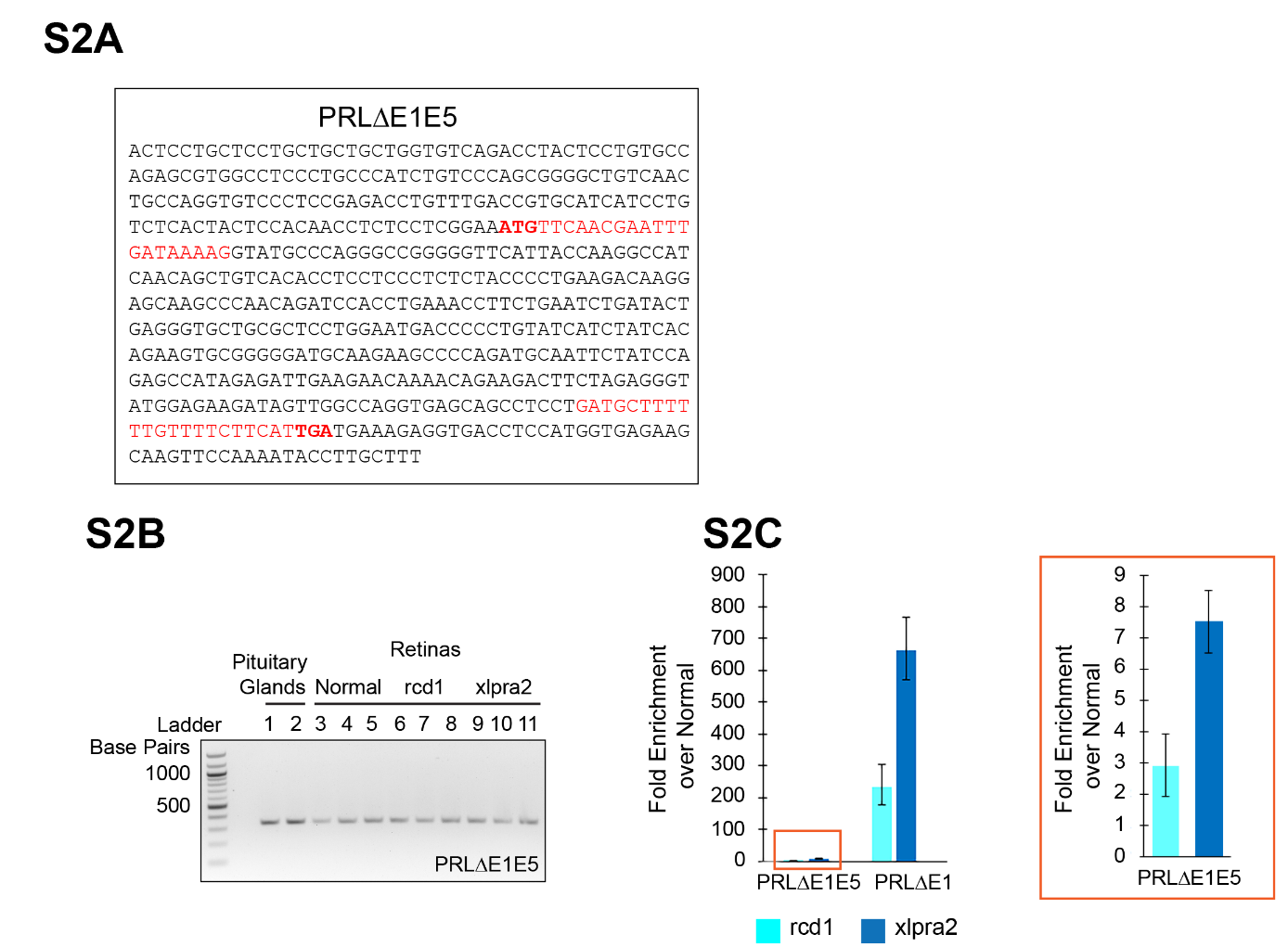


**Supplementary Figure S2:** Identification of a second short *PRL* isoform (PRLΔE1E5) expressed in normal and mutant retinas, as well as the pituitary gland and other tissues. **A)** The sequence for the PRLΔE1E5 lacking the first exon and ends within the fourth intron. The presumptive translational start and end sites, and the primer sequences are highlighted in red. **B)** PRLΔE1E5 is expressed in pituitary gland (n=2 dogs) and in normal and mutant retinas (n=3 dogs each). **C)** PRLΔE1E5 is upregulated in the mutant retinas to a much smaller extent compared to PRLΔE1.

**Supplementary Table 1A:** Summary of dogs used for this study

| **Dog ID** | **Phenotype** | **Age (weeks)** | **Gender** | **Organ** | **Experiment** |
| --- | --- | --- | --- | --- | --- |
| CEACMI | Normal | 24 | F | Eye | OS: RNA extraction, qPCR, PCR, 5’RACE |
| CEACID | Normal | 24 | F | Eye | OS: RNA extraction, qPCR, PCR, 5’RACE |
| CEACIV | Normal | 24 | F | Eye | OS: RNA extraction, qPCR, PCR |
| CGBCHK | Normal | 26 | F | Eye | OS: Western blot analysis; mass spectrometry  OD: OCT embedding without fixation, RNA-ISH |
| CGBCHL | Normal | 26 | F | Eye | OD: OCT embedding without fixation, RNA-ISH |
| CGBCHN | Normal | 26 | F | Eye | OS: Western blot analysis  OD: OCT embedding without fixation, RNA-ISH |
| 2149 | rcd1 affected | 22 | F | Eye | OD: OCT embedding without fixation, RNA-ISH  OS: RNA extraction, qPCR, PCR, 5’RACE |
| 2150 | rcd1 affected | 22 | F | Eye | OD: OCT embedding without fixation, RNA-ISH  OS: RNA extraction, qPCR, PCR, 5’RACE |
| 2151 | rcd1 affected | 22 | F | Eye | OD: OCT embedding without fixation, histology, immunohistochemistry  OS: RNA extraction, qPCR, PCR |
| 2225 | rcd1 affected | 22 | M | Eye | OS: Western blot analysis; mass spectrometry |
| 2226 | rcd1 affected | 22 | F | Eye | OS: Western blot analysis |
| Z478 | xlpra2 affected | 41 | F | Eye | OD: OCT embedding without fixation, RNA-ISH  OS: RNA extraction, qPCR, PCR, 5’RACE |
| Z479 | xlpra2 affected | 41 | F | Eye | OD: OCT embedding without fixation, RNA-ISH  OS: RNA extraction, qPCR, PCR, 5’RACE |
| Z480 | xlpra2 affected | 41 | F | Eye | OD: OCT embedding without fixation, histology, immunohistochemistry  OS: RNA extraction, qPCR, PCR |
| Z598 | xlpra2 affected | 41 | F | Eye | OS: Western blot analysis; mass spectrometry |
| Z599 | xlpra2 affected | 41 | F | Eye | OS: Western blot analysis |
| N324 | Normal | 63 | F | Pituitary gland | RNA extraction, PCR, 5’RACE |
| N323 | Normal | 63 | F | Pituitary gland | Western blot analysis |
| E1075 | Normal | 97 | F | Multiple (as listed in methods) | PCR |

**Supplementary Table 1B:** Archival OCT embedded retinal tissue used for RNA-ISH

| **Dog ID** | **Phenotype** | **Age (weeks)** | **Gender** |
| --- | --- | --- | --- |
| SPF | Normal | 4 | - |
| K617 | Normal | 8 | M |
| WB-E239 | Normal | 13 | M |
| RC702 | Normal | 24 | M |
| WB-T60 | Normal | 38 | - |
| 7148 | Normal | 165 | - |
| D347 | Normal | 354 | M |
| 1733 | rcd1 affected | 4 | M |
| 1913 | rcd1 affected | 5 | M |
| 1914 | rcd1 affected | 6 | M |
| 1907 | rcd1 affected | 7 | M |
| 1916 | rcd1 affected | 8 | F |
| 1742 | rcd1 affected | 12 | F |
| 1743 | rcd1 affected | 24 | F |
| Z201 | xlpra2 affected | 4 | M |
| Z203 | xlpra2 affected | 6 | F |
| Z405 | xlpra2 affected | 7 | F |
| Z251 | xlpra2 affected | 8 | M |
| Z219 | xlpra2 affected | 12 | F |
| Z226 | xlpra2 affected | 16 | F |
| Z204 | xlpra2 affected | 20 | M |

**Supplementary Table 2:** List of human neuroretinal tissue

| **Sample ID** | **Eye tissue location** | **Age** | **Sex** | **Time of death to collection** |
| --- | --- | --- | --- | --- |
| 2015-09-5876 | Temporal | 81 | F | 2.59 hours |
| 2015-07-4126 | Inferior | 75 | F | 3.44 hours |

**Supplementary Table 3:** List of primers used in this study

| **Experiment** | **Primer name** | **Figure** | **Primer sequences** | **Exon position** |
| --- | --- | --- | --- | --- |
| qPCR | PRL_qPCR FOR | 1B, 1C | 5’GTCATCCTGTCTCACTACATCC3’ | Exon 2 |
|  | PRL_qPCR REV | 1B, 1C | 5’CTGTTGATGGCCTTGGTAATG3’ | Exon 3 |
|  | PRL_qPCR_All_FOR | S2C | 5’CTATCCAGAGCCATAGAGATTG3’ | Exon 4 |
|  | PRL_qPCR_ΔE1_REV | S2C | 5’CTCTGATTCCAGGATGAACCT3’ | Exon 4-Exon 5 |
|  | PRL_qPCR_ΔE1E5_REV | S2C | 5’CATCAGGAGGCTGCTCAC3’ | Intron 4 |
|  | GAPDH FOR | 1B, 1C, S2C | 5'AACAGTGACACCCACTCTTC3' | Exon 4 |
|  | GAPDH REV | 1B, 1C, S2C | 5'CGGTTGCTGTAGCCAAATTC3' | Exon 5 |
| 5’RACE | PRL_GSP (dog) | 3B | 5'GATTACGCCAAGCTTTGGGGCTTCTTGCATCCCCCGCACT3' | Exon 4 |
|  | PRL_GSP (human) | 5B | 5'GATTACGCCAAGCTTGAAGGTCTTCATGGTGGATCTGTTGGGC3' | Exon 3 |
| PCR | PRL FOR (dog) | 4 A1, B1, C1 | 5'ATGGATAACAAAGGGTGGTC3' | Exon 1 |
|  | PRL REV (dog) | 4 A1, B1, C1, A2, B2, C2 | 5'GCAGTTGCTGTCGTAGAC3' | Exon 5 |
|  | PRLΔE1 FOR (dog) | 4 A2, B2, C2, S2A, S2B | 5'ATGTTCAACGAATTTGATAAAAG3' | Exon 2 |
|  | PRLΔE1E5 REV (dog) | S2A, S2B, | 5'ATGAAGAAAACAAAAAAGCATC3' | Intron 4 |
|  | RPS5 FOR (dog) | 4 B3, C3 | 5'ATGACCGAGTGGGAG3' | Exon 2 |
|  | RPS5 REV (dog) | 4 B3, C3 | 5'TCAACGGTTAGACTTGGC3' | Exon 6 |
|  | PRL FOR (human) | 6 C1 | 5’ATGAACATCAAAGGATCG3’ | Exon 1 |
|  | PRL REV (human) | 6 C1, C2 | 5’GCAGTTGTTGTTGTGGAT3’ | Exon 5 |
|  | PRLΔE1 FOR (human) | 6 C2 | 5’ATGTTCAGCGAATTCG3’ | Exon 2 |
|  | RPS5 FOR (human) | 6 C3 | 5’ATGACCGAGTGGGAG3’ | Exon 3 |
|  | RPS5 REV (human) | 6 C3 | 5’AGCGGTTGGACTTGG3’ | Exon 7 |
